# Loss of nephric augmenter of liver regeneration facilitates acute kidney injury via ACSL4‐mediated ferroptosis

**DOI:** 10.1111/jcmm.18076

**Published:** 2023-12-13

**Authors:** Lili Huang, Ling Zhang, Zheng Zhang, Fangyan Tan, Yixin Ma, Xujia Zeng, Dan Cao, Lili Deng, Qi Liu, Hang Sun, Bingbing Shen, Xiaohui Liao

**Affiliations:** ^1^ Department of Nephrology The Second Affiliated Hospital, Chongqing Medical University Chongqing China; ^2^ Department of Cell Biology and Genetics Chongqing Medical University Chongqing China; ^3^ Key Laboratory of Molecular Biology for Infectious Diseases, Ministry of Education, Institute for Viral Hepatitis The Second Affiliated Hospital of Chongqing Medical University Chongqing China; ^4^ Department of Nephrology Chongqing University Central Hospital, Chongqing Emergency Medical Center Chongqing China; ^5^ Kuanren Laboratory of Translational Lipidology, Centre for Lipid Research The Second Affiliated Hospital of Chongqing Medical University Chongqing China

**Keywords:** acute kidney injury, augmenter of liver regeneration, ferroptosis, lipid peroxidation, long‐chain‐fatty‐acid‐CoA ligase 4

## Abstract

Ferroptosis, characterized by lipid accumulation in intracellular compartments, is related to acute kidney injury (AKI), but the mechanism remains obscure. In our previous study, the protective effect of augmenter of liver regeneration (ALR) on AKI was not fully clarified. In this study, we established an AKI mouse model by knocking out proximal tubule‐specific ALR and an AKI cell model by inducing hypoxia, as well as enrolled AKI patients, to investigate the effects of ALR on ferroptosis and the progression of AKI. We found that *ALR* knockout aggravated ferroptosis and increased ROS accumulation and mitochondrial damage, whereas *ALR* overexpression attenuated ferroptosis through clearance of ROS and maintenance of mitochondrial morphology. Mechanistically, we demonstrated that ALR could directly bind to long‐chain‐fatty‐acid‐CoA ligase 4 (ACSL4) and further inhibit the expression of ACSL4 by interacting with certain regions. By resolution liquid chromatography coupled with triple quadruple mass spectrometry, we found that ALR could reduce the contents of polyunsaturated fatty acids, especially arachidonic acid. In addition, we showed that ALR binds to ACSL4 and attenuates oxylipin accumulation, exerting a protective effect against ferroptosis in AKI. Therefore, targeting renal ALR can attenuate ferroptosis and can offer a promising strategy for the treatment of AKI.

## 1 INTRODUCTION

Acute kidney injury (AKI), a common complication associated with high mortality and morbidity in critically ill patients, is characterized by a sharp decline in renal function and extensive proximal tubular cell death in a very short period of time (i.e. within 48 h).^1^, ^2^, ^3^ Ischemia–reperfusion (I/R) injury is the main cause of AKI.^4^, ^5^ Following I/R injury, renal function could recover partly within a few days of blood flow reversal.^6^, ^7^ However, if I/R injury is not reversed in a timely manner, then the kidneys are susceptible to chronic kidney disease and possibly end‐stage kidney failure. Presently, the outcomes of interventional treatments, including symptomatic and supportive therapy, as well as renal replacement for chronic AKI, have been disappointing.^8^, ^9^ Thus, it is important to define the mechanism of AKI and identify new therapies for AKI. A recent study has reported that aberrant biochemical and metabolic processes can trigger a variety of diseases, and they are the causative factors of inflammation.^10^ Compelling evidence indicates that kidney diseases are closely related to metabolic aberrations,^11^, ^12^, ^13^, ^14^, ^15^, ^16^ with kidney diseases causing metabolic aberrations, such as excessive lipid peroxidation, abnormal amino acid metabolism and aberrant glucose breakdown, especially in I/R injury‐induced AKI. Unfortunately, there are few studies addressing lipid peroxidation in AKI.

Ferroptosis, a unique iron‐dependent form of regulated cell death, is biochemically, morphologically and physiologically distinct from other forms of cell death, such as apoptosis, necrosis and autophagy.^17^, ^18^, ^19^ Lipid peroxides, a major source of free radicals, may trigger ferroptosis in the absence of sufficient NADPH and glutathione.^18^, ^20^ As a relatively new type of regulated cell death, several unique mitochondrial changes, such as the smaller than normal mitochondria, darker‐stained membranes and disorganization/reduction of mitochondrial crista, are considered to be the main morphological features distinguishing it from other cell death modes.^21^ Moreover, emerging evidence suggests that mitochondria play critical roles in ferroptosis^22^, ^23^, ^24^ and that ferroptosis is involved in AKI. For instance, inactivation of the ferroptosis‐associated gene, glutathione peroxidase 4 (GPX_4_), triggers AKI in mice,^25^, ^26^ whereas inhibition of ferroptosis by a pharmacological inhibitor can suppress proximal tubular cell death.^27^

Augmenter of liver regeneration (ALR), which is essential for respiration, is a growth‐promoting factor in the liver that was initially identified in the rat and reported to promote hepatocyte proliferation and liver regeneration.^28^ In addition, ALR is widely expressed by all mammalian tissues, including the kidneys.^29^ In our previous studies, we reported that ALR plays a protective role against oxidative injury by reducing reactive oxygen species (ROS) production and accumulation in renal proximal tubules.^21^, ^30^ The accumulation of lipid ROS is another hallmark of ferroptosis, although when, where and how they are generated during ferroptosis is not clear. Additionally, little is known about the relationship between excessive lipid peroxidation and ALR expression in ferroptosis.

In this study, we systematically analysed the differences in lipid composition after kidney‐specific deletion of the ALR gene in AKI. We also examined the relationship between ACSL4, a ferroptosis‐associated marker and ALR and concluded that ALR can alleviate lipid accumulation by inhibiting the ACSL4 pathway.

## 2 MATERIALS AND METHODS

### 2.1 Experimental model

Details are described in Figure S1. To generate the kidney‐specific ALR knockout mice, we conditionally knocked out the *ALR* gene (also known as GFER) using the Ggt1‐Cre/LoxP system. Briefly, ALR‐targeting vector with LoxP sites placed between exons 1 and 2 and before exon 3 of the *ALR* gene was constructed, and standard molecular techniques were employed to generate ALR^*flox/flox*^ mouse. To generate kidney‐specific ALR‐knockout mouse, hemizygous Ggtl‐Cre transgenic mice were first crossed with ALR^*flox/flox*^ mice, ALR^*flox/+*^‐Ggtl‐Cre offsprings were then crossed with ALR ^*flox/flox*^ mice to generate mice with following genotypes: ALR^*flox/flox*^‐Ggtl‐Cre, ALR^flox/+^‐Ggtl‐Cre, ALR^*flox/flox*^. The mice were maintained on a mixed C57BL/6 background.

### 2.2 Establishment of the ischemic AKI model in mice

All experiments were performed in accordance with the National Institutes of Health Guide for the Care and Use of Laboratory Animals, and all procedures were approved by the Chongqing Medical University Animal Care and Use Committee (approval no. 2018,146). Bilateral ischemic AKI was induced as previously described.^31^ Briefly, mice (C57BL/6 background, 6–8 weeks old, 18–22 g body weight) were anaesthetised with 60 mg/kg of sodium pentobarbital in 0.9% NaCl (stock concentration, 5 mg/mL) by intraperitoneal injection. The core body temperature was monitored with a rectal probe (HP‐30, Beijing Cinotech Co., China) attached to a temperature control system and maintained at 36.6–36.8°C (optimal temperature, 36.7°C ± 0.1°C). Bilateral flank incisions were made to expose the kidneys, and renal ischemia was induced for 22 min using micro‐clamps and micro‐clips (RS‐5420/RS‐5410, Roboz, UK). The micro‐clamps were removed, and the restoration of blood flow was visually confirmed. All mice were euthanized at 1, 2 and 3 days after renal ischemia. Blood and kidney tissues were collected.

### 2.3 Collection of blood and kidney tissue, and histological analysis of human kidney tissues

The study involving humans was approved by the Institutional Review Board of the Second Affiliated Hospital of Chongqing Medical University (approval no. 2018,146). Blood and kidney tissues were collected. The adjacent noncancerous tissues and the AKI tissues were used as the normal group (*n* = 6) and the AKI group (*n* = 6), respectively. The adjacent noncancerous tissues were collected from patients who underwent magnetic resonance imaging and pathological examination at the Second Affiliated Hospital of Chongqing Medical University. All patients provided written informed consent prior to surgery. The AKI patients were classified according to renal function as described in *Kidney Disease: Improving Global Outcomes*. All samples were removed aseptically and frozen in liquid nitrogen until use. Serum creatinine, serum blood urea nitrogen (BUN) and the glomerular filtration rate were measured with an automatic biochemical analyser.

### 2.4 Transmission electron microscopy

The kidney tissues were collected, minced into 1‐mm^3^ fragments and fixed in 2.5% glutaraldehyde fixative at 4°C. On the following day, the tissues were postfixed in 1% OsO_4_, dehydrated through a graded alcohol series at room temperature, and embedded in resin. The blocks were sectioned, and 80‐nm continuous sections were stained with 2% uranium acetate and observed under a transmission electron microscope (Hitachi, Japan).

### 2.5 High‐throughput RNA sequencing and informatic analysis

Total RNA was isolated with TRIzol Reagent (Invitrogen, China) according to the manufacturer's instructions. RNA quality, purity and quantity were assessed by Shanghai NovelBio Bio‐Pharm Technology Co., Ltd. The samples were processed with an Illumina HiSeq X Ten Sequencing System. The differentially expressed genes (DEGs) were filtered using the significant threshold value (*p*‐value), fold change (FC) and the false discovery rate (FDR). A total of 5042 DEGs were identified between the control and AKI groups (Table S1). Genes with *p*‐values <0.05 were identified as DEGs. A total of 130 DEGs were identified between control and ALR KO mice after I/R injury (Table S3). Genes with |log2FoldChange| >1 and *p*‐values <0.01 were identified as DEGs. Differences in pathways were identified using the Student's *t*‐test after correcting for multiple hypotheses and accepting pathways with a 5% FDR cutoff. All data were uploaded to the NCBI GEO public database (accession no. GSE 212678).

### 2.6 Detection of ROS in cells and tissues

For cells, the ROS levels were detected by dihydroethidium (BestBio, China). After H/R injury, the cells were harvested and incubated with dihydroethidium (1:1000) for 30 min at 37°C in the dark. The cells were washed with phosphate‐buffered saline and analysed by flow cytometry. For tissues, the ROS levels were detected by Cy3 (Sigma, USA). After H/R injury, the kidney sections were incubated with Cy3 (1:500) for 30 min at 37°C in the dark. The cell nuclei were counterstained with DAPI. The cells were viewed under a fluorescent microscope (Nikon Eclipse C1, Japan).

### 2.7 Establishment a hypoxia/reoxygenation (H/R) model in HK‐2 cells

Human kidney proximal tubular epithelial (HK‐2) cells were cultured in Dulbecco's Minimum Essential Medium F12 (Gibco, USA) supplemented with 10% fetal bovine serum (Moregate, Australia) and 1% penicillin–streptomycin (Invitrogen, USA) in an atmosphere of 5% CO_2_ at 37°C. To induce H/R injury, the HK‐2 cells were cultured in serum‐free medium overnight, followed by serum‐free and glucose‐free medium in an atmosphere of 94% N_2_, 5% CO_2_ and 1% O_2_ for 6 h, as previously described.^21^, ^32^ Subsequently, the HK‐2 cells were transferred to an atmosphere of 5% CO_2_ and cultured in a complete medium for 12 h.

### 2.8 Western blotting

The cells were washed, and mitochondrial proteins were extracted using the Mitochondrial Kit (Beyotime Biotech Co., Ltd., China) according to the manufacturer's instructions. The protein concentration was determined using the BCA Protein Assay (KenGen Biotech, China). The proteins were separated by 15% sodium dodecyl sulfate‐polyacrylamide gel electrophoresis (SDS‐PAGE) and transferred onto polyvinylidene membranes. After blocking in 5% nonfat milk in Tris‐buffered saline–Tween‐20 for 2 h at room temperature, the membranes were incubated with specific primary antibodies overnight at 4°C. Subsequently, the membranes were washed three times with Tris‐buffered saline–Tween‐20 and incubated with the indicated secondary antibodies for 1 h at room temperature. The ECL Kit (Beyotime Biotech Co., Ltd.) was used for detecting immunoreactive proteins. The antibodies were as follows: NGAL (1:1000, cat no. ab216462m, Abcam, UK), KIM‐1 (1:1000, cat no. NBP1‐76701, Novus, USA), GPX_4_ (1:8000, cat no. ab125066, Abcam), ACSL4 (1:5000, cat no. ab155282, Abcam), ALR (1:1000, cat no. abx129857, Abcam), COX IV (1:1000, cat no. 4844, Cell Signalling Technology, USA) and xCT (1:10000, cat no. ab175186, Abcam).

### 2.9 Quantitative real‐time polymerase chain reaction (qRT‐PCR) analysis

RNA was extracted from cells and tissues using the Total RNA Purification Kit (Biyuntian, China). Approximately 1 μg of RNA was reverse transcribed into cDNA using the Reverse Transcription Kit (TaKaRa, Japan), and qRT‐PCR was performed using the SYBR Premix Kit (TaKaRa). The primer sequences are listed in Table S6.

### 2.10 Co‐immunoprecipitation

HK‐2 cells were lysed in lysis buffer, and the protein concentration was determined as previously indicated. The protein lysates (1.5 mg) were incubated with the indicated antibodies at a protein: antibody ratio of 500:1 overnight at 4°C on a rotator. The antibodies were as follows: anti‐ALR polyclonal antibody (cat no. 11293, Proteintech, USA) and anti‐ACSL4 polyclonal antibody (cat no. ab155282, Abcam). Anti‐IgG (cat no. 3900, Cell Signalling Technology) was used as the negative control. On the following day, 100 μL of a 20% slurry of agarose beads (Abcam) was washed three times with lysis buffer and added to the samples for 2 h at 4°C on a rotator. The immunocomplexes were centrifuged at 2817 *g* for 5 min at 4°C, and the supernatants were washed three times with lysis buffer and centrifuged to yield the “input” sample. The immunocomplexes were combined with an equivalent volume of 1× SDS‐PAGE sample buffer and heated at 95°C for 5 min. Western blotting was performed as described above.

### 2.11 Immunofluorescent cell staining

To determine whether ALR and ACSL4 colocalize in mitochondria, the MitoTracker Red CMXRos Kit (Beyotime Biotech Co., Ltd.) was used to label viable mitochondria. The HK‐2 cells were seeded in confocal microscopy dishes and allowed to adhere to the substrate overnight. After H/R injury, the cells were washed with phosphate‐buffered saline three times and stained with trimethylrhodamine, methyl ester, for 20 min, followed by fixation with 4% paraformaldehyde. The cells were washed, permeabilized, blocked with 0.5% BSA and incubated overnight at 4°C with primary antibodies (ACSL4, 1:100 or ALR, 1:50). On the following day, the cells were incubated with a fluorescein isothiocyanate (FITC)‐ or a tetramethylrhodamine isothiocyanate (TRITC)‐conjugated secondary antibody for 60 min at room temperature in the dark. The cell nuclei were counterstained with DAPI. The cells were observed under a confocal laser‐scanning microscope (Nikon A1R + Confocal Microscope, Nikon, Japan).

### 2.12 siRNA and plasmids

The ACSL4 siRNA target sequence was 5′‐GCAAUAAUCCUGCUAUGGAtt‐3′, and the siRNA negative control (NC) sequence was 5′‐UUCUCCGAACGUGUCACGUdTdT‐3′. The siRNAs were purchased from Shanghai GeneBio (China), and they were used at a concentration of 50 nM. The pReceiver‐Lv105 plasmid and empty vector (negative control) were purchased from Shanghai GeneBio. The cells were transiently transfected with Lip2000 Transfection Reagent (Thermo Fisher Scientific, USA) according to the manufacturer's instructions. The transfection efficiency was determined by qRT‐PCR analysis. The sequence of ACSL4 for transient gene knockdown is provided in Table S6. The sequence of the expression clone, as well as its verification, is provided in Figure S4A–C.

### 2.13 Lentivirus transfection and stable cell clone establishment

The lentiviral‐based small hairpin RNA (shRNA) and the ALR overexpression construct were purchased from GeneChem (China). The HK‐2 cells were infected with lentiviruses with an MOI 6. The full‐length ALR gene (GFER, NM_005262) was synthesized using a human cDNA library. All the sequences (Table S3) were cloned into a lentiviral vector. The order of the elements in the ALR overexpression construct was Ubi–MCS–3FLAG–SV40–EGFP–IRES–puromycin. At 35% confluence, the HK‐2 cells were infected with lenti‐ALR‐EGFP (designated as ALR overexpression, ALR‐OE) or lenti‐EGFP (designated as vector). After 72 h, the transfection efficiency was assessed by fluorescent microscopy, and the cells were selected for 2 weeks with puromycin (3 μg/mL, Sigma‐Aldrich, USA) to produce a stable cell line for subsequent assays. The verification of the lentivirus is presented in Figures S3A–D and S4D.

### 2.14 Protein–protein docking

The ClusPro server is a widely used tool for protein–protein docking studies and binding affinity analyses.^33^ ACSL4 was designated as the ligand, whereas ALR was designated as the receptor. The ligand was rotated 70,000 times, and for each rotation, the ligand was translated in x, y and z planes relative to the receptor on a grid. The translation with the highest score was selected. Of the 70,000 rotations, 1000 rotation–translation combinations with the lowest score were selected, and greedy clustering of these ligand positions with a 9 Å C‐alpha RMSD radius was performed to identify the ligand positions with the most “neighbours,” that is, cluster centres. The top 10 cluster centres were retrieved and visually inspected, and the intermolecular contacts from the most probable poses were further evaluated. After analysing the docked structures and interface residues, the molecular images were generated by PyMOL (www.pymol.org).

### 2.15 UPLC‐QqQ‐MS/MS analysis of oxylipins

The separation and quantification of oxylipins from HK‐2 cells were performed using UPLC coupled with 6460 QqQ‐MS/MS (Agilent Technologies, Germany). Briefly, the cells were sonicated twice and stored at −40°C for 1 h. After centrifugation (15 min, 13,523 *g*, 4°C), 480 μL of the supernatant was transferred to an Eppendorf tube, and 320 μL of water was added. After vortexing for 30 s, the sample was further purified with solid‐phase extraction (SPE). The SPE cartridges were equilibrated (methanol/water,1:1). After loading the supernatant, the cartridge was washed with 1 mL of 5% MeOH/H_2_O. The flow‐through fraction was discarded, and the samples were eluted with 1 mL of MeOH. The eluent was evaporated to dryness under a gentle stream of nitrogen and reconstituted in 100 μL of 30% ACN/H_2_O. The reconstituted sample was vortexed for 30 s, homogenized at 60 Hz for 4 min, and sonicated for 5 min in an ice‐water bath. After centrifugation (1 min, 13,523 *g*, 4°C), the sample was transferred to an EP tube with a filter membrane. After centrifugation (15 min, 13,523 *g*, 4°C), the clear supernatant was subjected to UPLC‐MS/MS analysis. The precision of the quantitation was measured as the relative standard deviation (RSD), which was determined by injecting analytical replicates of a QC sample. The accuracy of the quantitation was measured as the analytical recovery of the QC sample was determined. The per cent recovery was calculated as [(mean observed concentration)/(spiked concentration)] × 100%.

### 2.16 Statistical analysis

Data were presented as the mean ± standard error of the mean (SEM). GraphPad Prism 8.0 Software (GraphPad Software, Inc., USA) was used for statistical analysis. Differences between the two groups were assessed by an unpaired Student's *t*‐test. For multiple group comparisons, one‐way anova, followed by Tukey's post hoc test, was applied. Significant differences were considered at *p*‐values <0.05.

## 3 RESULTS

### 3.1 I/R injury induces ferroptosis in AKI

**FIGURE 1:**
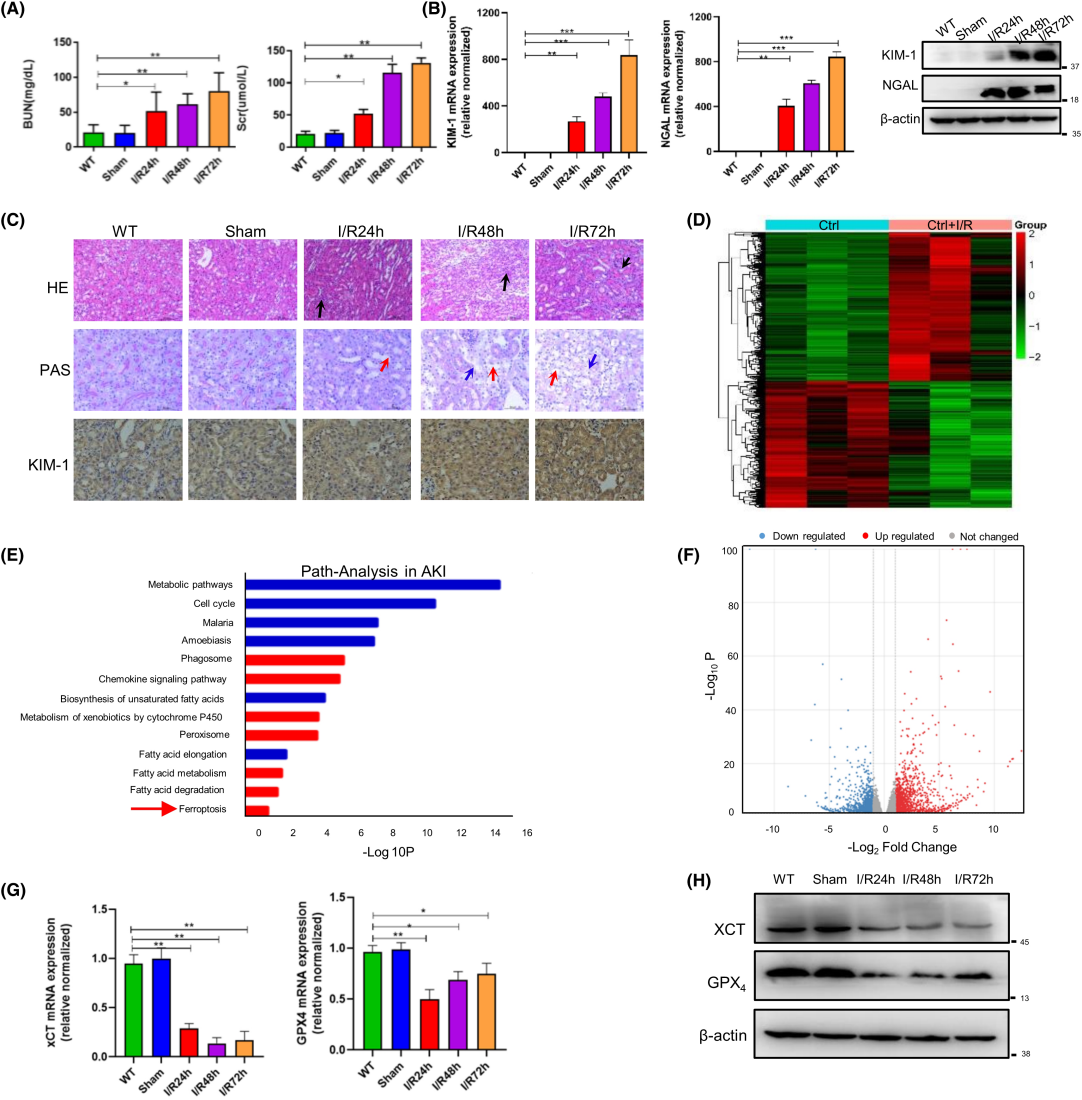
Ischemia–reperfusion injury‐induced AKI involves ferroptosis. (A) Baseline serum creatinine and blood urea nitrogen levels (*n* = 6). Blood and kidney tissues were collected at 24, 48 and 72 h after I/R injury. Data represent mean ± SD of at least three independent experiments, *p < 0.05, versus control; **p < 0.01, ***p < 0.001 versus the indicated groups. (B) KIM‐1 and NGAL expression in kidneys as determined by quantitative real‐time polymerase chain reaction (qRT‐PCR) and western blotting. (C) Representative haematoxylin–eosin‐ and periodic acid–Schiff‐stained kidney sections (magnification, 20×), and KIM‐1 expression in kidneys after I/R injury. Inflammatory cell infiltration is indicated by black arrows, brush border loss is represented by red arrows and tubular basement membrane damage is indicated by blue arrows. (D) Heatmap of differentially expressed genes. Red represents upregulated genes, and green represents downregulated genes. (E) Gene set enrichment analysis of up‐regulated (red) and down‐regulated (blue) differentially expressed pathways in control mice and mice after 24 h of I/R injury (*n* = 3). (F) Volcano plot of all genes. Red dots represent up‐regulated genes, and blue dots represent down‐regulated genes that exceeded the FDR cutoff. The y‐axis represents −log_10_ of FDR, and the x‐axis represents –log_2_ fold change. (G) xCT, GPX_4_ expression in kidneys, as determined by qRT‐PCR (*n* = 3). (H) xCT, GPX_4_ and ALR expression in kidneys, as determined by western blotting (*n* = 3).

I/R injury is the main cause of AKI. Thus, the bilateral renal I/R injury model was used.^31^ We evaluated the pathological changes, as well as renal function, after 22 min of ischemia and different times of reperfusion. With prolonged reperfusion time, we observed extensive pathological changes and deteriorating renal function. Compared with wild‐type mice, serum creatine and BUN levels were elevated in mice with prolonged reperfusion time (Figure 1A). The mRNA and protein levels of biomarkers for proximal tubule injury, namely kidney injury molecule‐1 (KIM‐1) and neutrophil gelatinase‐associated lipocalin (NGAL), were elevated in AKI mice (Figure 1B). In addition, we observed brush border loss, tubule flattening and inflammatory cell infiltration (Figure 1C), indicating that I/R injury can induce AKI in mice. To explore the pathways involved in AKI, we performed RNA sequencing (RNA‐seq) using mouse kidney tissues. RNA‐seq data of control mice and I/R injury‐induced mice can be found in Table S1. The heatmap showed significant differences in gene expression between non‐AKI and AKI mouse groups (Figure 1D). In gene set enrichment analysis, several pathways were associated with AKI, with the top three up‐regulated pathways related to cell cycle progression, malaria and amoebiasis and the top down‐regulated pathway related to metabolism (Figure 1E). We used a volcano plot to compare differentially expressed genes (DEGs) in control and I/R injury‐induced mice and plotted the overall distribution of DEGs (Figure 1F). Ferroptosis was the main signalling pathway identified in this analysis. Ferroptosis is controlled by glutathione peroxidase 4 (GPX_4_). Cysteine availability can limit glutathione biosynthesis and cystine import, which are required by the cystine/glutamate antiporter system x_c_^−^.^34^ Thus, we examined xCT and GPX_4_ expression in the kidneys of I/R injury‐induced mice (Figure 1G,H), and the results revealed that I/R injury can induce ferroptosis.

### 3.2 ALR is highly expressed and ALR deficiency exacerbates AKI

**FIGURE 2:**
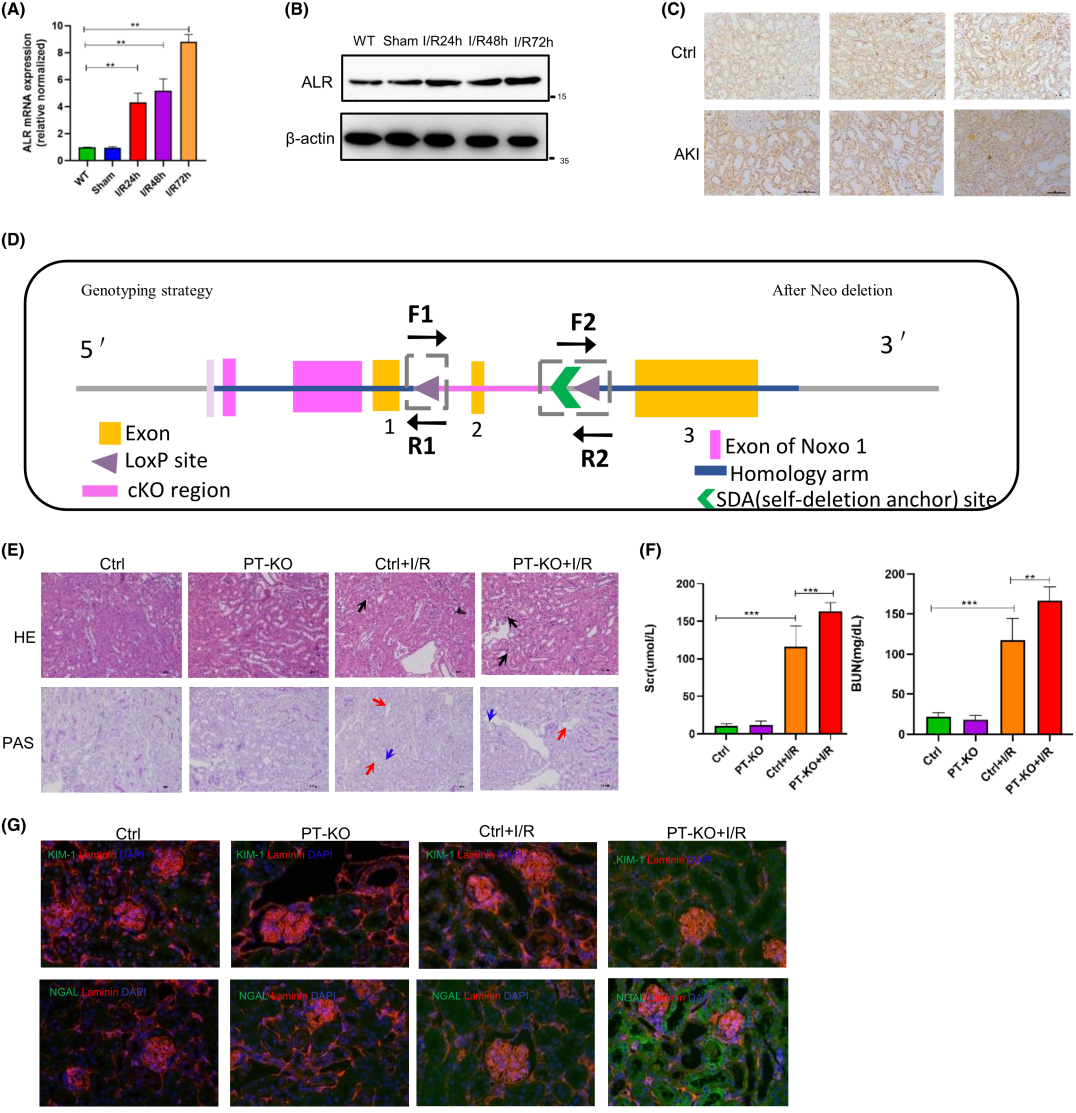
Elevated ALR expression in AKI and ALR knockdown exacerbates ischemia–reperfusion‐induced AKI. (A) ALR expression in mouse kidneys, as determined by quantitative real‐time polymerase chain reaction (*n* = 6). (B) ALR expression in mouse kidneys, as determined by western blotting (*n* = 5). (C) Representative immunohistochemical staining images of ALR in human AKI samples. (D) Strategy of ALR kidney‐specific ALR knockout mice and schematic diagram of a targeted mutation in the ALR gene in mouse ES cells. (E) Haematoxylin–eosin‐ and periodic acid‐Schiff‐stained kidney sections of ALR KO mice (*n* = 6). Magnification, 20×. Inflammatory cell infiltration is indicated by black arrows, brush border loss is represented by red arrows and tubular basement membrane damage is indicated by blue arrows. (F) Biochemical analysis of renal function in ALR KO and wild‐type mice (*n* = 6). *p < 0.05, versus control; **p < 0.01, *** *p* < 0.001 versus the indicated groups. (G) Representative KIM‐1‐ and NGAL‐stained kidney sections. Scale bar = 20 μm.

Using RNA‐seq data, we screened the differential expression of ALR to investigate its expression in I/R injury‐induced AKI mice and AKI patients. Mice were studied at 24, 48 and 72 h after ischemia–reperfusion injury, and ALR expression was increased compared with the control (Figure 2A,B). These results were confirmed by immunohistochemistry in AKI patients (Figure 2C). The AKI patient information is presented in Figure S2 and Table S2. To understand the role of ALR in ferroptosis in AKI, we generated a conditional KO mouse (C57BL/6 background) in which the ALR gene was deleted in proximal tubular cells by the Cre‐Loxp system (Figure 2D). The primer sequences for genotyping are listed in Figure S1A. By cross‐breeding the ALR floxed mouse with the Ggt1‐Cre mouse, we obtained the ALR^flox/flox^/Ggt1‐Cre mouse (PT‐KO mouse). ALR^flox/flox^ mice were used as the control (Figure S1B). ALR expression was high in the liver; thus, we examined ALR expression in this organ by qRT‐PCR and western blotting. The protein was abundantly expressed in the liver. In the kidneys, ALR expression in PT‐KO mice was decreased compared with control mice (Figure S1C,D,G). Approximately 8 weeks after birth, there were no significant differences in body weight and the kidney/body weight ratio between PT‐KO and wild‐type mice (Figure S1E,F). To examine the pathological changes of ALR knockout on renal tubule damage in AKI, we performed haematoxylin–eosin and periodic acid–Schiff staining. ALR KO mice were euthanized after 24 h of I/R injury, at which time the epithelial cells of proximal tubules were injured. Compared with control mice, haematoxylin–eosin and periodic acid–Schiff‐stained kidney sections demonstrated extensive damage of proximal tubular epithelial cells in PT‐KO mice (Figure 2E). Furthermore, after 24 h of reperfusion, we observed brush border loss, tubule flattening, and epithelial cell sloughing in PT‐KO mice compared with control mice. Renal function deteriorated in PT‐KO mice as evidenced by increased serum creatine and BUN levels (Figure 2F), but there were no significant differences in these parameters between PT‐KO mice and wild‐type mice in the absence of I/R injury. These results were confirmed by immunofluorescent staining (Figure 2G).

### 3.3 ALR protects against I/R injury‐induced ferroptosis in vivo and in vitro

**FIGURE 3:**
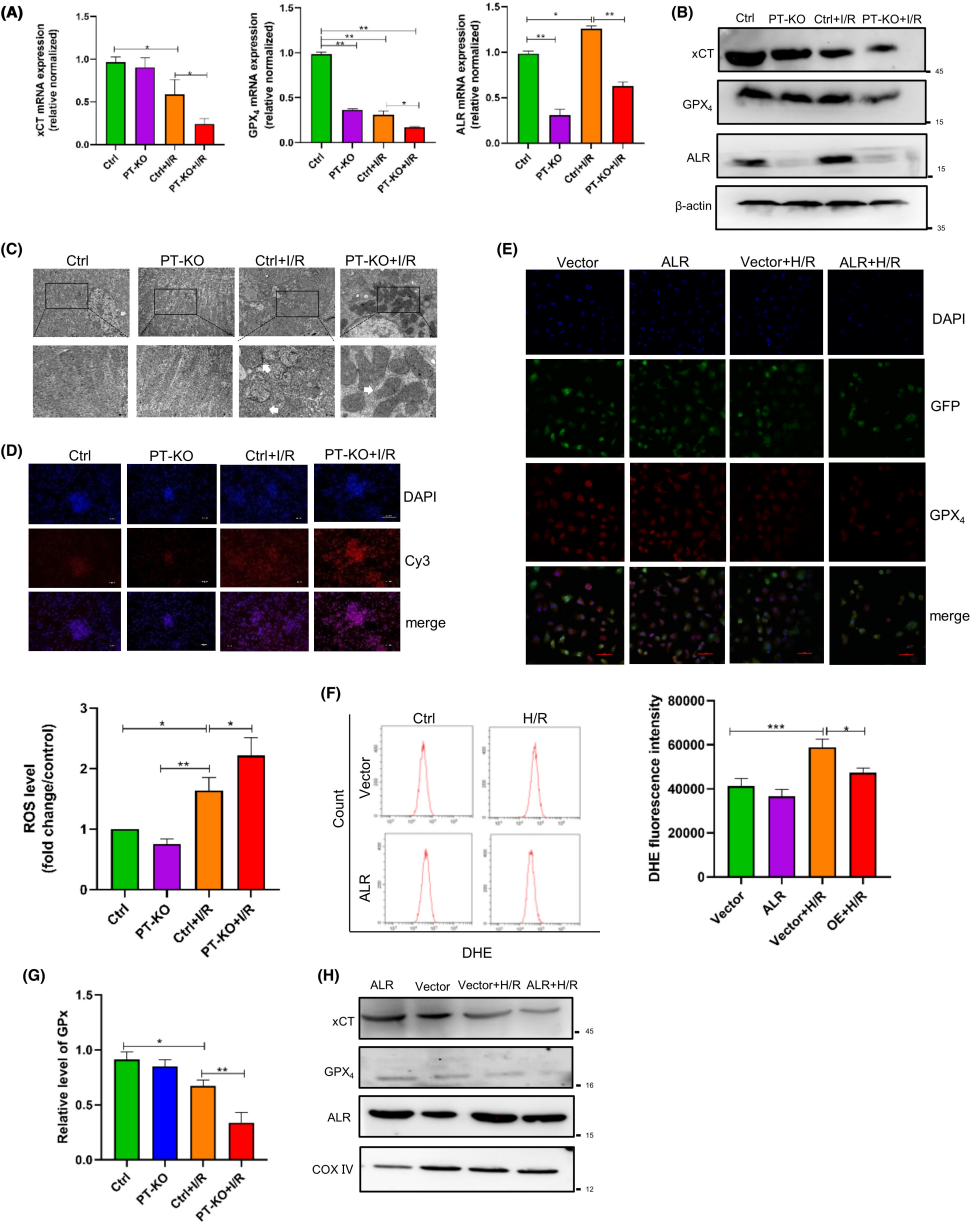
ALR protects against I/R injury‐induced ferroptosis in vitro and in vivo. (A) xCT, GPX_4_ and ALR expression in ALR KO and wild‐type mice as determined by quantitative real‐time polymerase chain reaction (*n* = 6). (B) xCT, GPX_4_ and ALR expression in mouse kidneys. (C) Representative transmission electron microscopy images of mouse kidney tissue after I/R injury. Magnification, 10,000× or 30,000×. Scale bars = 2 μm or 500 nm, respectively. White arrows indicate mitochondrial cristae disappearance and outer membrane rupture. ROS level was quantified and normalized with the control group. (D) ROS levels in the kidneys were measured by hydro‐Cy3 staining. Stained cells were viewed by confocal laser‐scanning microscopy. (E) GPX_4_ expression and localization after ALR overexpression in HK‐2 cells exposed to H/R injury. GPX_4_ localization was examined by confocal laser‐scanning microscopy. Scale bar = 20 μm. (F) ROS levels in HK‐2 cells were measured by dihydroethidium. Stained cells were analysed by flow cytometry (*n* = 3). *p < 0.05, versus control; **p < 0.01, *** *p* < 0.001 versus the indicated groups. (G) Relative GPX content. *p < 0.05, versus control; **p < 0.01, *** *p* < 0.001 versus the indicated groups. (H) Western blot confirmed xCT, GPX_4_ and ALR expression in mitochondria after ALR overexpression in H/R injury‐induced cells.

To evaluate the role of ALR in ferroptosis, we examined the changes in the expression of ferroptosis‐related molecules. After I/R injury, the mRNA and protein levels of xCT and GPX_4_ in PT‐KO mice decreased after I/R injury compared with wild‐type mice (Figure 3A,B). Morphological changes in mitochondria are the most representative feature of ferroptosis, so we used transmission electron microscopy to observe mitochondrial morphology. After I/R injury, proximal tubule cells in PT‐KO mice had smaller than normal mitochondria with darker‐stained mitochondrial membranes and disorganized mitochondrial crista compared with wild‐type mice (Figure 3C). Given that ferroptosis is promoted by excessive ROS accumulation in cells, we examined ROS levels in PT‐KO mice. In PT‐KO mice, the ROS levels in the kidneys were elevated after I/R injury (Figure 3D). To examine the role of ALR in vitro, we transiently overexpressed ALR by lentiviral infection and successfully established H/R injury in HK‐2 cells (Figure S3A–D). We also observed GPX_4_ expression in the nucleus and cytoplasm (Figure 3E). H/R injury reduced GPX_4_ expression, whereas ALR overexpression promoted GPX_4_ expression in HK‐2 cells. Next, we detected ROS levels in HK‐2 cells after H/R injury. In H/R injury‐induced cells, the ROS levels were elevated, whereas ALR overexpression reduced the ROS levels in HK‐2 cells (Figure 3F). We also measured GPX activity and found that it was significantly reduced in PT‐KO mice after I/R injury compared with wild‐type mice (Figure 3G). Given that ferroptosis associates with mitochondrial changes,^23^ we examined the cellular levels of mitochondrial proteins associated with ferroptosis. After H/R injury, xCT and GPX_4_ expression was significantly increased in cells overexpressing ALR compared with those overexpressing the empty vector (Figure 3H). These results indicate that cell‐specific knockout of ALR aggravates ferroptosis in proximal tubule cells and promotes AKI in mice, whereas overexpression of ALR in HK‐2 cells alleviates ferroptosis in proximal tubule cells.

### 3.4 ACSL4 plays a key role in ferroptosis

**FIGURE 4:**
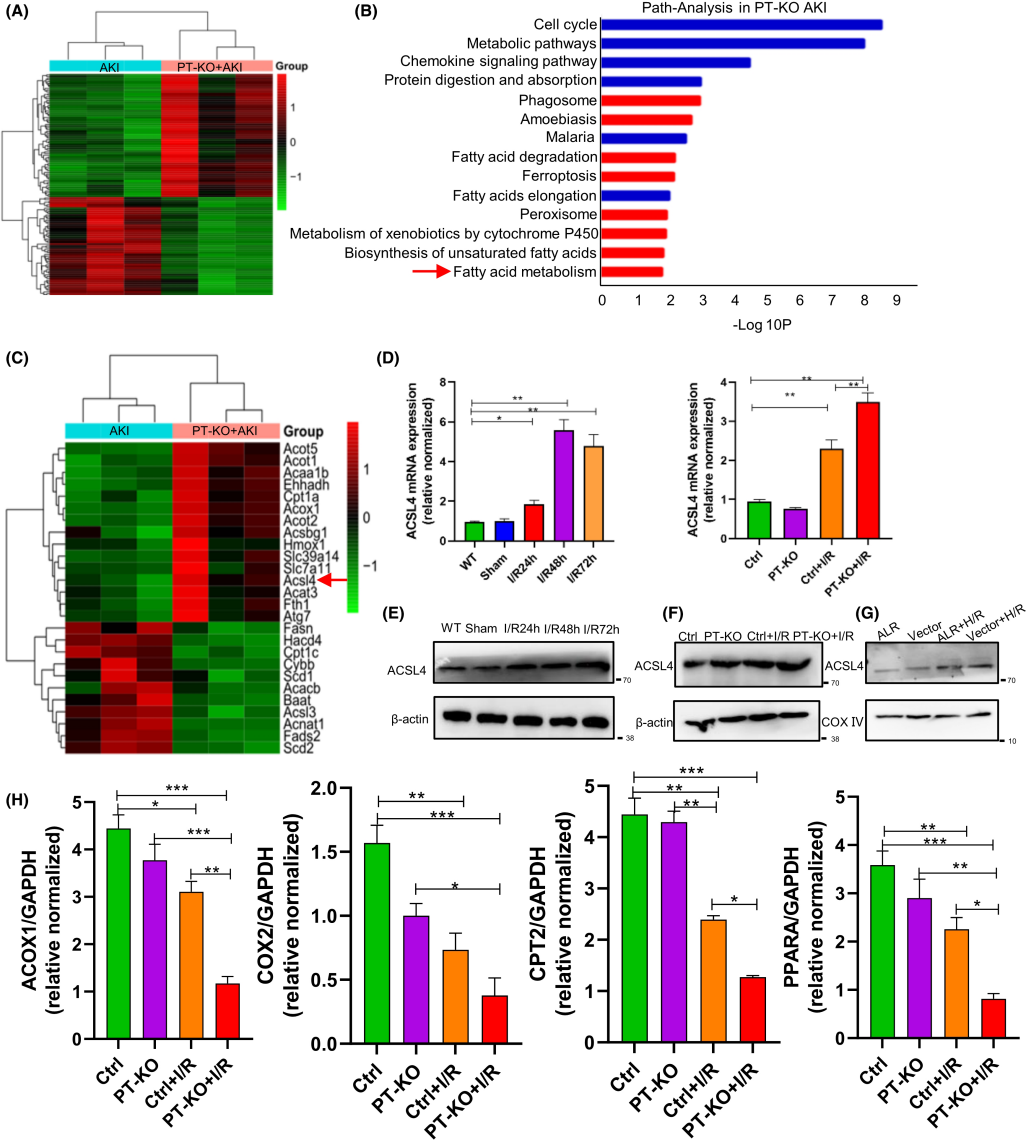
ALR knockout drives expression of fatty acid‐associated genes. (A) Supervised hierarchical clustering identifies two groups after deep sequencing of RNA. Heatmap was prepared using a diverging scale, in which up‐regulated genes are coloured red and down‐regulated genes are coloured green. The first group represents AKI mice, and the second group represents PT‐KO AKI mice. These clusters were further classified into AKI (light green) and PT‐KO AKI (orange) (*n* = 3). (B) Pathway analysis of differentially expressed genes from (A) was performed. The red arrow indicates fatty acid metabolism. (C) Heatmap of differentially expressed genes related to fatty acid synthesis and ferroptosis between AKI and PT‐KO ALR mice. The red arrow indicates ACSL4 (*n* = 3). (D) ACSL4 expression in vivo (*n* = 6) as determined by quantitative real‐time polymerase chain reaction in AKI mice with or without ALR knockout. *p < 0.05, versus control; **p < 0.01 versus the indicated groups. (E–G) ACSL4 expression in vivo and in vitro (mitochondrial fractions from HK‐2 cells) as determined by western blotting in AKI mice and HK‐2 cells after hypoxia–reoxygenation. (H) Fatty acid oxidation enzyme expression. *p < 0.05, versus control; **p < 0.01, versus the indicated groups.

To further explore the effects of ALR on ferroptosis, transcriptome sequencing was performed using kidney tissues from PT‐KO mice. A heatmap revealed significant differences in gene expression between AKI and PT‐KO AKI mice (Figure 4A). In gene set enrichment analysis, fatty acid metabolism pathways were identified (Figure 4B). RNA‐seq data of mice with AKI and PT‐KO mice with AKI can be found in Table S3. Lipid peroxidation promotes ferroptosis, so we identified 26 genes related to fatty acid accumulation and ferroptosis (Figure 4C) from AKI and ALR KO mouse groups. The detailed genes are listed in Table S4. Next, we examined ACSL4 expression in wild‐type and ALR KO mice after I/R injury‐induced AKI expression and observed that ACSL4 expression increased significantly in ALR KO mice after I/R injury compared with wild‐type mice (Figure 4D–F). Furthermore, ALR overexpression could further rescue ACSL4 expression in vitro (Figure 4G), indicating that ACSL4 is a biomarker of ferroptosis in AKI^35^, ^36^ and that ALR regulates ACSL4 expression in AKI. In the presence of ferrous iron or lipoxygenase, ALR catalyses unsaturated fatty acids within cell membranes, which causes lipid peroxidation and ferroptosis. Thus, we examined the levels of fatty acid oxidation genes in the kidneys. We observed that the levels of enzymes and regulators of fatty acid oxidation were reduced after I/R injury compared with the control (Figure 4H), indicating that ALR plays a key role in ferroptosis by inhibiting fatty acid oxidation.

### 3.5 ALR binds ACSL4 to prevent ferroptosis

**FIGURE 5:**
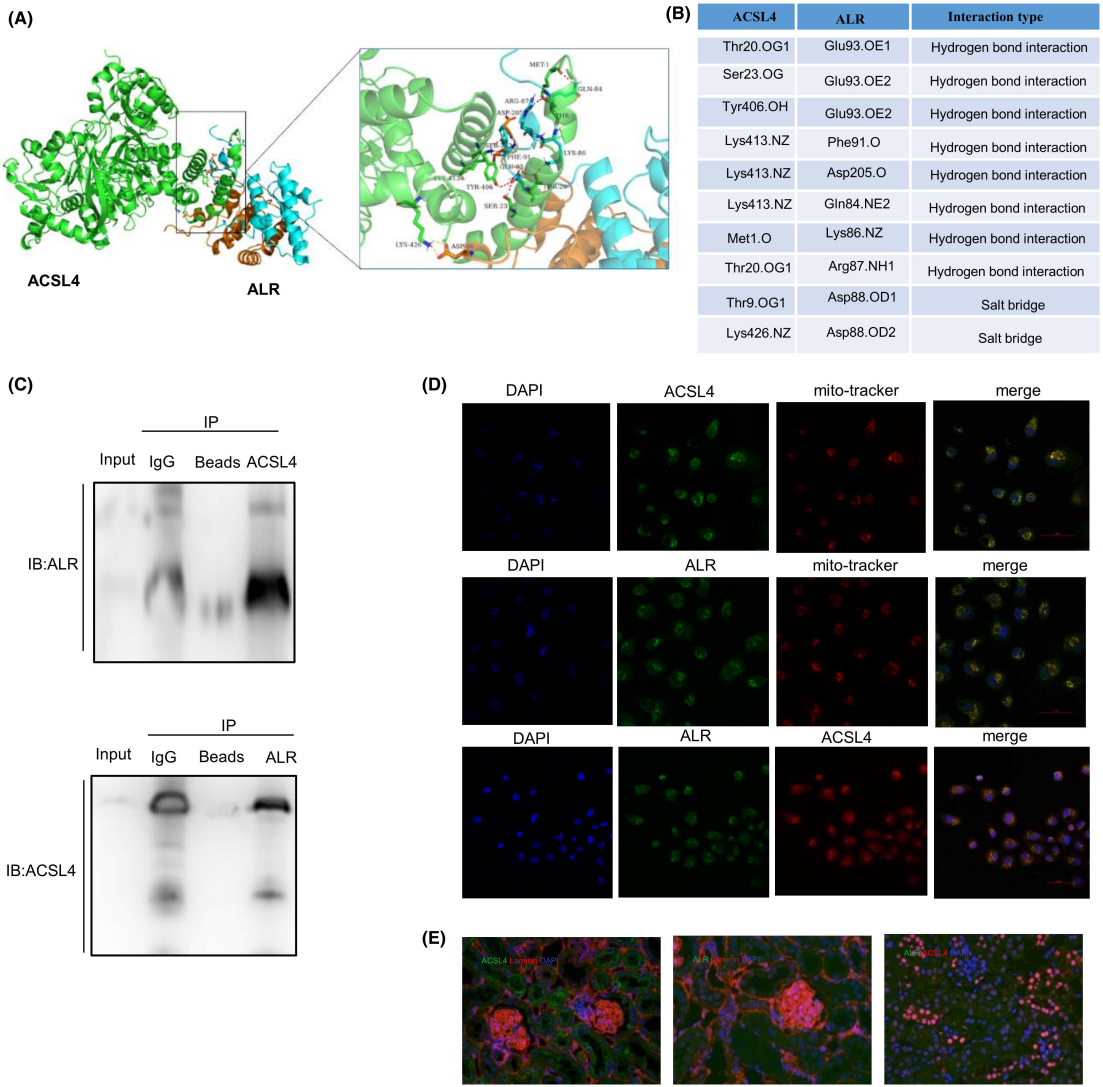
ALR interacts with ACSL4. (A) Surface binding model of ALR with ACSL4. ALR is coloured cyan (chain A) and orange (chain B), and ACSL4 is coloured green. Red dashes represent hydrogen bonds, and yellow dashes represent salt bridges. (B) Specific sites of contact between ALR and ACSL4. (C) Co‐immunoprecipitation of endogenous ALR and ACSL4 in human proximal tubule cells. (D) ALR and ACSL4 colocalize in the mitochondria of HK‐2 cells by confocal laser‐scanning microscopy. Scale bar = 50 μm. (E) ALR and ACSL4 colocalize in mouse epithelial cells of proximal tubules. Sections were co‐stained with anti‐laminin to outline the tubular area. Scale bar = 20 μm (*n* = 3).

Interactions between ALR and ACSL4 in publicly available protein–protein interaction databases have not been reported. To confirm whether ALR and ACSL4 interact, we identified the potential binding sites between these two proteins, and molecular docking was used to identify potential interacting residues based on geometric complementarity. Given that ALR is a dimer,^37^ we analysed the potential binding sites in the dimer. Interacting regions between ALR (https://www.pdbus.org/, PDB, ID3MBG) and ACSL4 (https://www.uniprot.org/, Uniprot‐O60488‐F1‐model) corroborated the available data on the existing domains (Figure 5A). The specific sites of contact between ACSL4 and ALR are shown in Figure 5B. There were eight hydrogen bonds and two salt bridges between ALR and ACSL4. Co‐immunoprecipitation verification of ALR and ACSL4 interaction in HK‐2 cells (Figure 5C). In addition, ALR and ACSL4 were co‐localized in mitochondria in HK‐2 cells (Figure 5D). In mice, these two proteins are mainly localized in the proximal tubules (Figure 5E).

### 3.6 Oxidation oxylipin of downstream ACSL4 reduced ferroptosis is regulated by ALR in AKI

**FIGURE 6:**
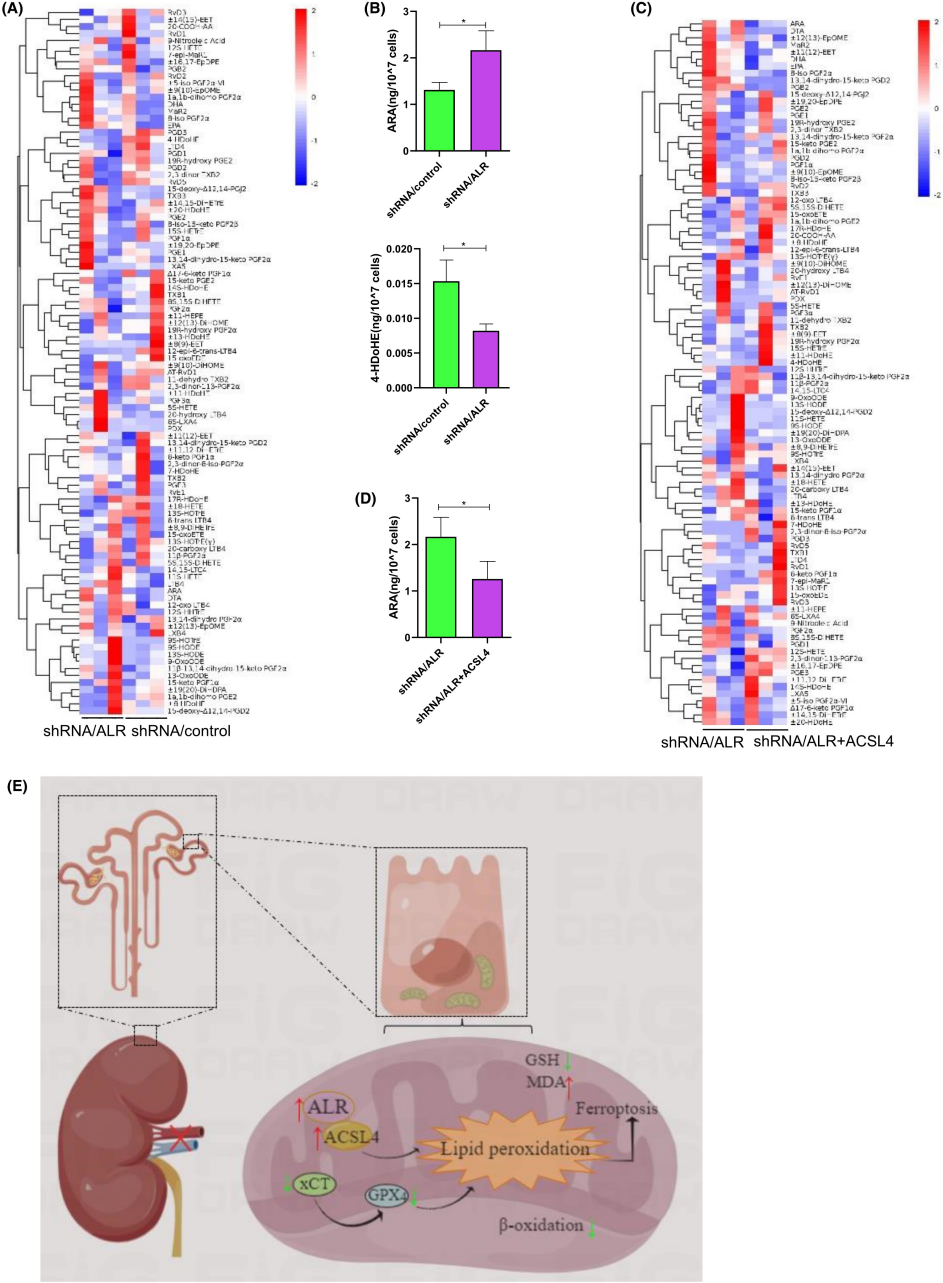
Information about oxylipin by UPLC‐QqQ‐MS/MS. (A) Targeted metabolomics profiling assays of oxylipin after lentivirus‐mediated shRNA interference of ALR expression. (B) Contents of 4‐HDoH4 and ARA (only statistically significant changes are displayed). (C) Targeted metabolomics profiling assays of oxidized lipids after inhibition of ALR expression through shRNA interference and/or ACSL4 overexpression plasmid transfection. (D) Content of ARA. *p < 0.05, versus control; **p < 0.01 versus the indicated groups. (E) Working model of ALR mediating mitochondrial intermembrane assembly by binding to ACSL4, thereby decreasing ferroptosis. Green arrows represent downregulation, whereas red arrows represent upregulation.

Ferroptosis is characterized by the accumulation of ROS and lipid peroxidation products. There are many different classes of cellular lipids, including monounsaturated fatty acids and polyunsaturated fatty acids. However, it is unclear whether these fatty acids are oxidized and whether they have roles in ferroptosis. To address these questions, we extracted and analysed lipid metabolites by UPLC‐QqQ‐MS/MS as well as analysed the oxylipin ratio between ACSL4‐knockdown and control samples to generate the lipidomic profiles. There were 48 lipid metabolites (17 down‐regulated and 31 up‐regulated) displaying changes after ACSL4 knockdown (Table S5 and Figure S4E). A volcano map showed the down‐regulation and up‐regulation of oxylipin (Figure S4F). As for the qualitative oxylipin profile, of the 48 oxylipins analysed, there were significant differences in six oxylipins between ACSL4‐knockdown and control samples (Figure S4G). To further examine the functions of ALR and ACSL4 in ferroptosis, ALR expression was silenced in HK‐2 cells by short hairpin interfering RNA sequences. The vector information and transfection efficiency of ALR and ACSL4 are shown in Figure S4A–D. Next, we performed targeted lipidomics and metabolomics profiling after interfering with ALR expression (Figure 6A) and found that the level of 5Z,8Z,11Z,14Z‐eicosatetraenoic acid (ARA) increased significantly compared with the control (Figure 6B). Next, we transfected shRNA/ALR cells with an ACSL4 overexpressing plasmid and detected oxidized lipids by targeted metabolomics. A heatmap showed the individual lipid species from targeted metabolomics profiling (Figure 6C). After overexpression of the ACSL4 plasmid, we examined the ARA level (oxylipin from EPA) and observed a significant increase (*p* < 0.05) in the shRNA/ALR group compared with the shRNA/ALR + ACSL4 group (Figure 6D). Taken together, these findings prove that interference of ACSL4 and/or ALR can reduce polyunsaturated fatty acids and that ALR mediates mitochondrial intermembrane assembly by binding to ACSL4 (Figure 6E), thereby decreasing ferroptosis.

## 4 DISCUSSION

Ferroptosis, a non‐apoptotic form of cell death, is a relatively recently discovered regulated cell death that can be triggered by excessive lipid peroxidation.^38^ An increasing number of studies indicate that ferroptosis is involved in I/R injury, as well as AKI.^39^ I/R injury is the most common cause of AKI, and the pathophysiology of I/R injury‐induced AKI can be summarized by hemodynamic alterations, epithelial cell injury and inflammation.^40^ Furthermore, ROS production has been implicated in kidney damage after I/R injury. ROS are the products of lipid oxidation, and the rapid increase in ROS production can overwhelm antioxidant defences and further aggravate injury. It is worth mentioning that epithelial cells are susceptible to I/R injury. Previously, we reported that ALR was a protective antioxidant molecule in the mitochondrial response to H/R injury‐induced oxidative stress in AKI.^41^ Another study also found that defects in ALR function lead to mitochondrial iron accumulation.^28^ It is worth mentioning that ALR is responsible for Fe‐S cluster transfer and mitochondrial membrane stability.^42^ Moreover, ferroptosis is involved in iron‐dependent, lipid peroxide accumulation. Here, we reveal the significance of ALR in I/R injury‐induced ferroptosis in the kidneys by RNA‐seq. We found that ALR expression was significantly increased in H/R injury‐induced HK‐2 cells, I/R injury‐induced AKI mouse models and AKI patients. At present, it is not known why the expression of ALR, a protective molecule, increases during AKI. We speculate that feedback mechanisms caused by high ROS stress may be involved in AKI. In addition, several reports demonstrate that ALR is a secreted cytokine.^43^, ^44^ To further elucidate the protective role of ALR on ferroptosis in I/R injury‐induced AKI, we used a kidney‐specific ALR knockout mouse model. We also established an AKI mouse model by inducing bilateral ischemia for 22 min^45^ and reperfusion for 24 h. Knockout of ALR causes serious pathological damage, and the mortality rate of ALR KO mice was approximately 50% (data not shown). Therefore, we chose 24 h after reperfusion as the time point for further study. Taken collectively, these findings provide indirect evidence for the protective effects of ALR.

To explore the mechanism of ALR protection against ferroptosis, we speculated that ALR may be interacting with proteins involved in ferroptosis. We base this conjecture on the fact that ALR is a mitochondrial intermembrane space protein and oxylipins are produced in mitochondria. Therefore, we identified the common genes between fatty acid oxidation and ferroptosis in PT‐KO mice, and ACSL4 was the only gene that was common to both processes out of 26 genes. ACSL4, a member of the acy1‐CoA synthetase family, is a biomarker and regulator of ferroptosis.^35^ ACSL4 is localized in mitochondria, and it prefers polyunsaturated fatty acids.^46^ In this study, we identified by docking analysis the potential binding sites between ALR and ACSL4. We also observed that ALR and ACSL4 were colocalized in mitochondria and epithelial cells of proximal tubules in mice. In addition, ACSL4 expression increased significantly in ALR KO mice, and ALR overexpression could further rescue ACSL4 expression in vitro. These findings suggest that ALR may regulate ACSL4 expression by binding to ACSL4.

Ferroptosis is a lipid peroxide‐driven form of cell death. In other words, ferroptosis is driven by the peroxidation of membrane lipids such as phospholipids and glycerol‐derived lipids.^47^ Membrane lipids contain polyunsaturated fatty acids that exert highly lethal effects upon lipid peroxidation. Interestingly, ACSL4 induces ferroptosis in liver cells via arachidonic acid (ARA) oxidation.^48^ Therefore, we examined the products of lipid oxidation by UPLC‐QqQ‐MS/MS. We also observed changes in arachidonic and eicosapentaenoic acids following H/R injury, which decreased ACSL4 expression. Specifically, interference of ALR reduced the levels of polyunsaturated fatty acids, especially ARA, and overexpression of ACSL4 inhibited the content of ARA. Membranes rich in ARA facilitate ferroptosis during cell death.^49^ Our results indicate that ALR specifically binds to ACSL4, which reduces the accumulation of oxylipins, especially polyunsaturated fatty acids, in proximal tubule cells.

In summary, we revealed that the protective role of ALR could be enhanced via regulated ACSL4, an important regulator of the sensitivity of ferroptosis to convert and control the accessibility of long polyunsaturated fatty acids required for metabolic processes. These results provide new insights on how mitochondria, key organelles of energy resources for proximal tubule cells, can be targeted to decrease the accumulation of oxidized lipids in I/R injury‐induced AKI.

## AUTHOR CONTRIBUTIONS

**Lili Huang:** Data curation (equal); methodology (lead); software (supporting); writing – original draft (lead). **Ling Zhang:** Conceptualization (lead); project administration (supporting); resources (supporting); supervision (supporting); validation (equal). **zheng Zhang:** Conceptualization (supporting); formal analysis (supporting); methodology (supporting); software (supporting); supervision (supporting); visualization (lead). **Fangyan Tan:** Data curation (equal); investigation (supporting); methodology (supporting); software (supporting). **Yixin Ma:** Methodology (supporting); validation (supporting); visualization (supporting). **Xujia Zeng:** Data curation (supporting); investigation (supporting); methodology (supporting). **Dan Cao:** Data curation (equal); methodology (supporting); writing – original draft (supporting). **Lili Deng:** Methodology (supporting). **Qi Liu:** Supervision (supporting); validation (equal); visualization (supporting). **Hang Sun:** Conceptualization (supporting); formal analysis (supporting); project administration (lead). **Bingbing Shen:** Funding acquisition (supporting); methodology (supporting); resources (supporting); visualization (supporting). **Xiaohui Liao:** Funding acquisition (lead); investigation (equal); supervision (lead).

## FUNDING INFORMATION

This work was supported by grants from the National Natural Science Foundation of China (81873604), Chongqing medical scientific research project (Joint project of Chongqing Health Commission and Science and Technology Bureau) (2022GDRC005), Chongqing Natural Science Foundation (CSTB2022NSCQ‐MSX0984), CQMU Program for Youth lnnovation in Future Medicine (W0173) and Chongqing Key Laboratory of Emergency Medicine Open Project (2022KFKT05).

## CONFLICT OF INTEREST STATEMENT

All authors declare no conflicts of interest.

## Supporting information


Figure S1.

Figure S2.

Figure S3.

Figure S4.

Table S1.

Table S2.

Table S3.

Table S4.

Table S5.

Table S6.

## Data Availability

Data will be made available upon reasonable request.
