# Supplementary figures and images for: Loss of nephric augmenter of liver regeneration facilitates acute kidney injury via ACSL4‐mediated ferroptosis
Source: J Cell Mol Med. 2023 Dec 13;28(3):e18076. doi: 10.1111/jcmm.18076 (PMC10844764; doi:10.1111/jcmm.18076)

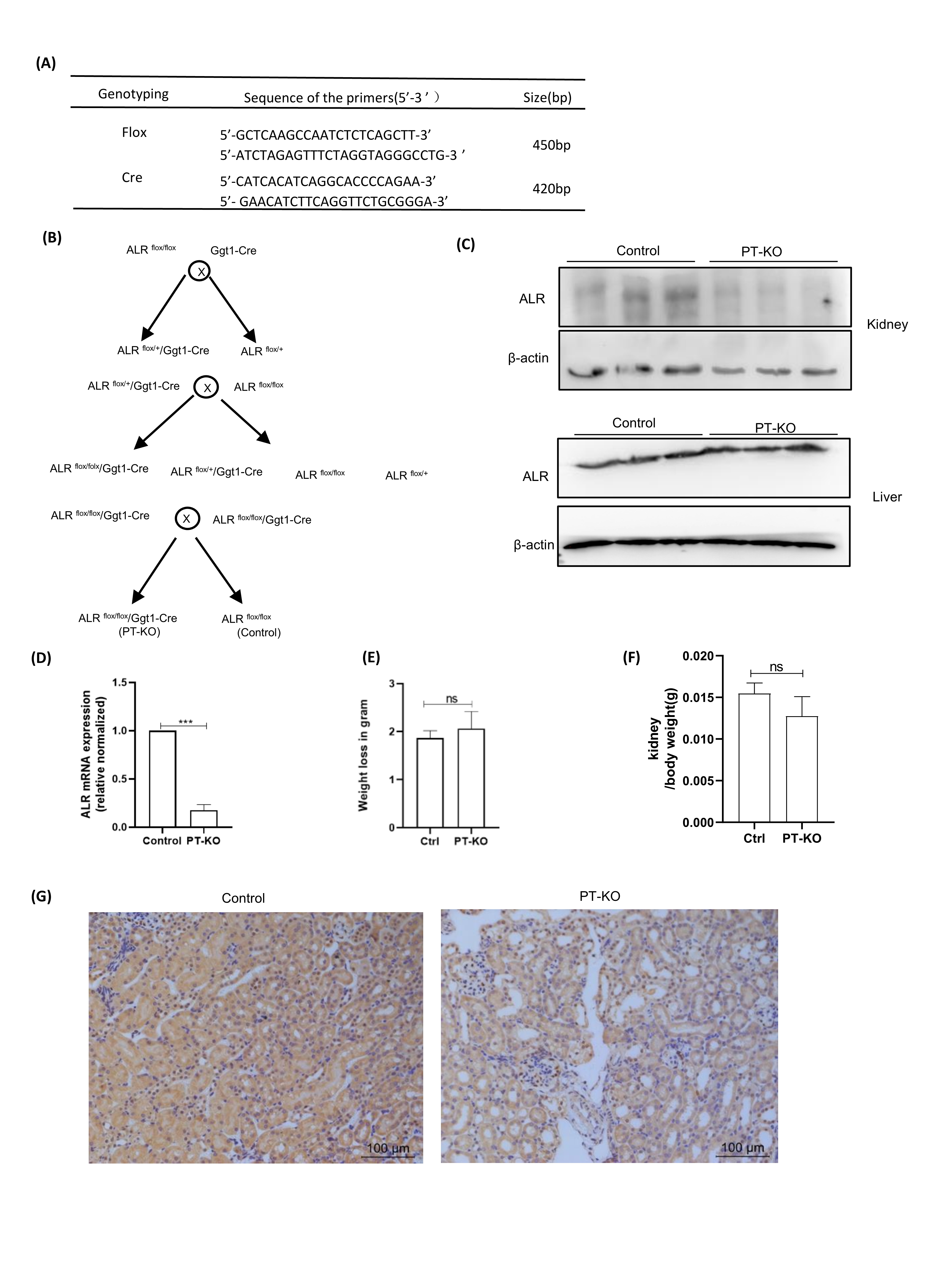

Supplement: Supplementary file 1 — Figure S1. [file JCMM-28-e18076-s008.tiff]

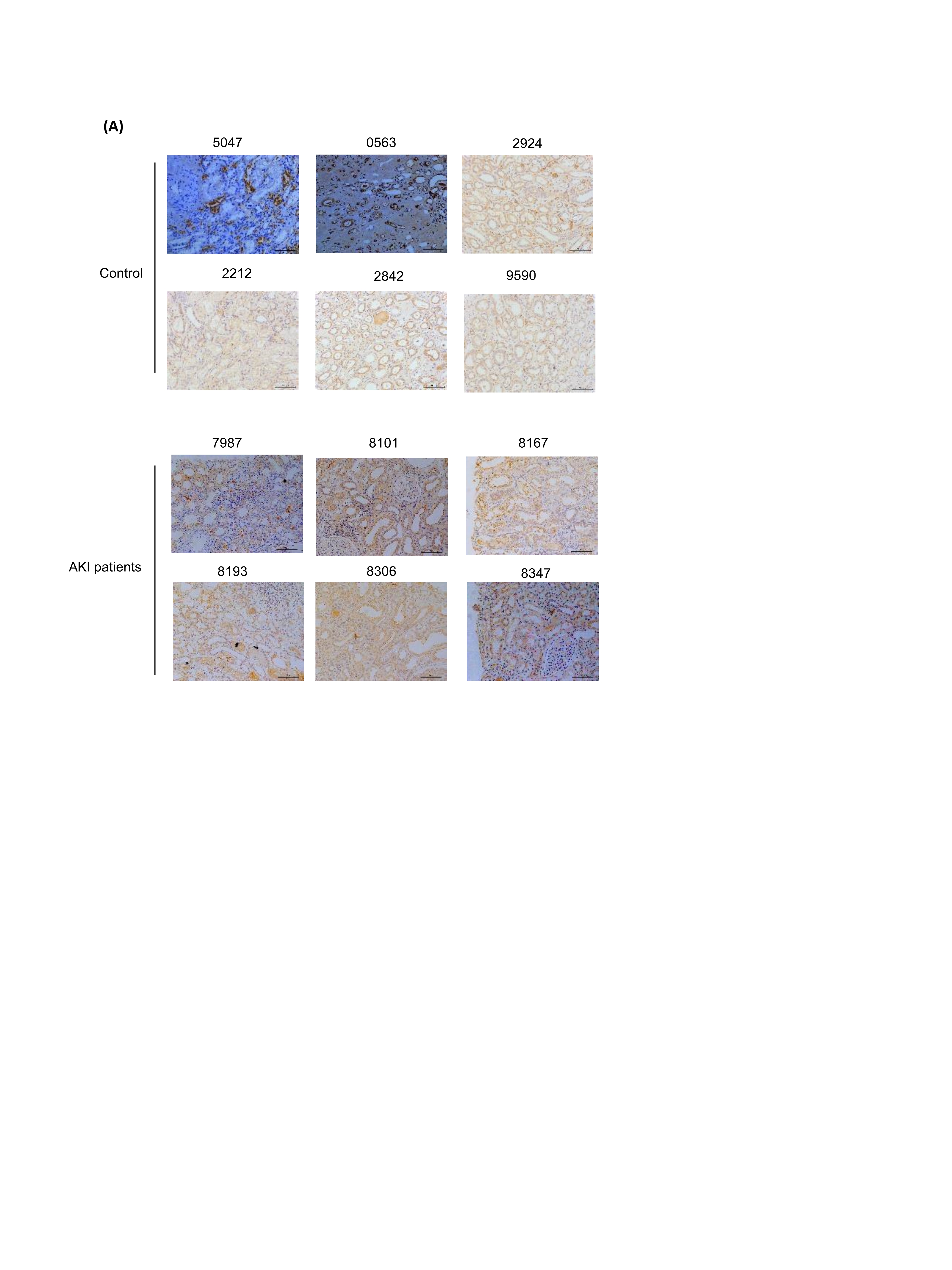

Supplement: Supplementary file 2 — Figure S2. [file JCMM-28-e18076-s009.tiff]

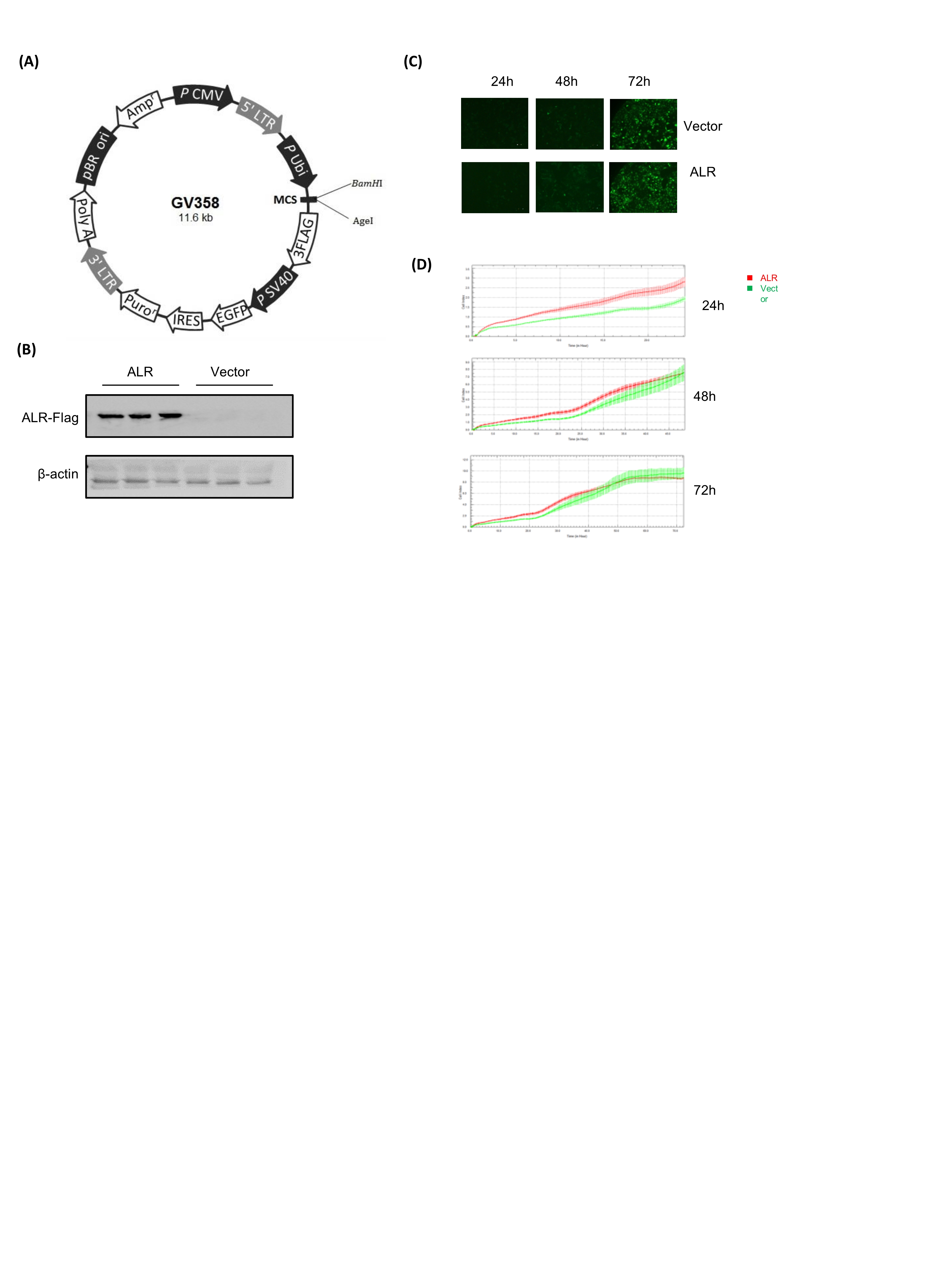

Supplement: Supplementary file 3 — Figure S3. [file JCMM-28-e18076-s004.tiff]

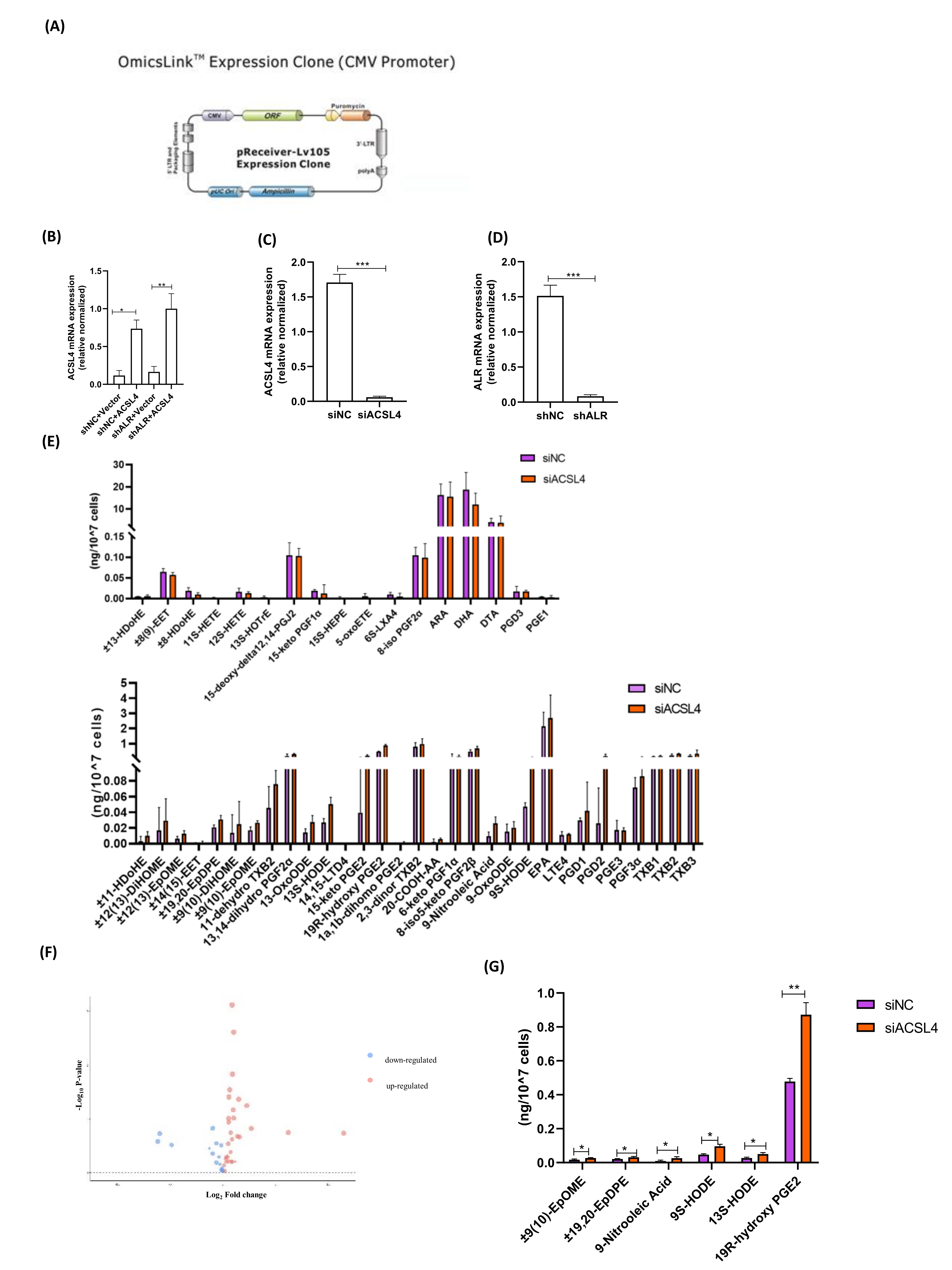

Supplement: Supplementary file 4 — Figure S4. [file JCMM-28-e18076-s001.tiff]
