# Supplementary material for: Loss of nephric augmenter of liver regeneration facilitates acute kidney injury via ACSL4‐mediated ferroptosis
Source: J Cell Mol Med. 2023 Dec 13;28(3):e18076. doi: 10.1111/jcmm.18076 (PMC10844764; doi:10.1111/jcmm.18076)
Supplement: Supplementary file 6 — Table S2. [file JCMM-28-e18076-s006.docx]

5047/2842/2924/2212/9590/0563

17987/ 8101/8167/8193/8306/8347

113.1 ± 23.2

acute tubular necrosis/

interstitial nephritis/

glomerulonephritis/

crescent formation

**Pathological findings**

**ID**

**AKI**

**normal tissues**

Female

Male

2

4

3

3

**Age(year)**

**Gender**

**Race**

HAN

HAN

**Scr(μmol/L)**

**BUN(mmol/L)**

**eGFR（ml/min）**

59.3±15.3

6

6

16.4 ± 5.8

7.85 ± 2.56

14.5 ± 7.7

63.9 ± 20.4

444.2 ± 175.3

35.5±6.9

-
