# Supplementary material for: Loss of nephric augmenter of liver regeneration facilitates acute kidney injury via ACSL4‐mediated ferroptosis
Source: J Cell Mol Med. 2023 Dec 13;28(3):e18076. doi: 10.1111/jcmm.18076 (PMC10844764; doi:10.1111/jcmm.18076)
Supplement: Supplementary file 8 — Table S4. [file JCMM-28-e18076-s003.docx]

**Same**

**N**

**ACSL4/ACSL3**

**20**

**8**

**Fatty acids**

**Ferroptosis**

Acaa1b/Acox1/Acot2

Fads2/Scd2/Hacd4

Scd1/Baat/Acot5

Acnat1/Acot1/Cpt1c

Cpt1a/Fasn/Acsbg1

Acat3/Acacb/ACSL4

ACSL3 /Ehhadh

Hmox1/Cybb

Slc39a14/Fth1

Slc7a11/Atg7

Acsl4/Acsl3
